# Severe community‐acquired pneumonia compared to severe community‐acquired *Acinetobacter baumannii* pneumonia in Reunion Island: A retrospective study

**DOI:** 10.1111/tmi.14067

**Published:** 2024-12-04

**Authors:** Giacomo Rotini, Axel de Mangou, Agathe Combe, Amelie Renou, Chloe Combe, Radj Cally, Marie Lagrange‐Xelot, Nicolas Allou, Guillaume Miltgen, Charles Vidal

**Affiliations:** ^1^ Department of intensive care medicine Felix Guyon University Hospital Saint‐Denis Reunion France; ^2^ Department of infectious and tropical disease Felix Guyon University Hospital Saint‐Denis Reunion France; ^3^ Department of microbiology, Felix Guyon University Hospital, UMR PIMIT, CNRS 9192, INSERM U1187, IRD 249 University of Reunion Island Saint‐Denis Reunion France

**Keywords:** *Acinetobacter baumannii*, Reunion Island, severe community‐acquired pneumonia, tropical region

## Abstract

*Acinetobacter baumannii* (*Ab*) has emerged in the last decades as a cause of community‐acquired pneumonia (CAP) in tropical and subtropical regions. We previously conducted the first investigation on this topic in France with a case series of severe CAP‐*Ab* in Reunion Island over an eight‐year period. In the present work, we aim to highlight the specific aspects of CAP‐*Ab* by comparing our case series with an historical cohort (PAC_RUN), obtained by retrospective chart review (2016–2021) of severe community‐acquired pneumonia cases on Reunion Island, in which CAP‐*Ab* was ruled out. During the study period, eight CAP‐*Ab* cases were identified, giving an incidence of 0.1 cases per 100,000 people/year, and an incidence of 16.5 cases per 100,000 people/year for non‐*Ab‐*related CAP (*n* = 761). By comparing with non‐*Ab‐*related CAP, patients had more excessive alcohol use (75% vs. 25.6%, *p* = 0.005) and lower body mass index (21 vs. 24 kg/m^2^, *p* = 0.004). Six cases (75%) of CAP‐*Ab* occurred during the rainy season (*p* = 0.06). Mortality was higher (62.5% vs. 24.3%, *p* = 0.02) and time to death was shorter (median 2 days vs. 7, *p* = 0.009) in the CAP‐*Ab* group. Bacteraemic pneumonia was strongly associated with CAP‐*Ab* (62.5% vs. 15.7%, *p* = 0.004). Significant differences were found in the need for renal replacement therapy (75% vs. 17.2%, *p* < 0.001), catecholamine use (100% vs. 54.5%, *p* = 0.01) and use of invasive mechanical ventilation (100% vs. 62.7%, *p* = 0.03). Also, in the proportion of severe acute respiratory distress syndrome (62.5% vs. 23.2%, *p* = 0.02), septic shock (100% vs. 40.6%, *p* < 0.001), and cardiogenic shock (87.5% vs. 15.9%, *p* < 0.001). Compared to severe non‐*Ab‐*related CAP, severe CAP‐*Ab* is characterised by higher mortality, associated with a high frequency of multiple organ failure. Excessive alcohol consumption and malnutrition seem to be risk factors. To improve outcomes, broader spectrum antibiotic therapy must be immediately proposed when CAP‐*Ab* is suspected.

## INTRODUCTION

*Acinetobacter baumannii* (*Ab*) is a Gram‐negative, non‐fermenting coccobacillus that has been well‐described in nosocomial infections but also in traumatic wounds (e.g., on battlefields or following natural disasters). *Acinetobacter baumannii* are also described in community‐acquired infections, especially in tropical regions [1]. Regarding this last example, cases of community‐acquired *Ab* pneumonia (CAP‐*Ab*) are more common [2]. They typically occur during the rainy season among middle‐aged men with a history of excessive alcohol use and are characterised by a fulminant course with a high lethality of around 60% [3, 4]. Reunion Island (France), located in the Southwest Indian Ocean, has a tropical climate and a rainy season that lasts six months, from November to April [5]. Pneumonia in tropical regions differs from temperate climates in terms of pathogens and clinical syndrome [6]. The aim of this work was to compare severe CAP‐*Ab* with severe non‐*Ab‐*related CAP, in Reunion Island, to call up the specific aspects of CAP‐*Ab*.

## MATERIALS AND METHODS

### Sample selection

In a previous publication [7], we reported eight cases of CAP‐*Ab* by retrospective chart review of all patients from October 2014 to October 2022 who had an *Ab* isolate from blood and/or respiratory samples upon hospital admission, meeting the criteria for CAP [8] and having been admitted to one of the two ICUs of the Reunion Island university hospitals. For control patients, we used a retrospective cohort (PAC_RUN) from Chenu de Thuet [5] and Combe [9], from which CAP‐*Ab* was ruled out. They performed a retrospective chart review of all adult patients diagnosed with severe CAP hospitalised in Reunion Island's two ICUs between January 2016 and October 2021 (Saint‐Denis and Saint Pierre university hospitals).

### Definitions

Community‐acquired pneumonia was defined, according to the guidelines of the French Ministry of Public Health, as pneumonia acquired outside of the hospital and the diagnosis of which had to be made within 48 h of hospital admission [8]. Diagnosis was based on a group of symptoms and signs (fever >38°C, cough, expectoration, chest pain, dyspnoea, and signs of invasion of the alveolar space) associated with a new lung infiltrate on chest x‐ray or computed tomography scan [8]. Severe pneumonia was defined as any patient hospitalised in the ICU with one major criterion or ≥3 minor criteria as recommended by the American Thoracic Society [10]. Excessive alcohol use was defined by an AUDIT‐C score ≥10.

### Microbiological investigations

Bacteriological examination of blood cultures and respiratory samples were systematically performed (sputum samples on non‐intubated patients and tracheal or bronchoalveolar lavage on intubated patients). Identification was performed with Gram staining followed by culturing with definitive micro‐organism identification by MALDI‐TOF mass spectrometry. Antibiotic susceptibility testing was assessed by disc diffusion method or MIC determination by gradient strips. Interpretation was performed using the European Committee on Antimicrobial Susceptibility Testing (EUCAST) recommendations. Viral identification was performed using the multiplex real‐time PCR, Allpex (TM) Respiratory Seegene Panel.

### Statistical analysis

Data are expressed as total number (percentage) of categorical variables and as median [interquartile range] of continuous variables. We used a Mann–Whitney *U* test to compare continuous variables and a Fisher exact test for categorical variables. We chose not to use the Pearson‐Chi squared test in the first analysis considering the limited number of patients in the CAP‐*Ab* group. Survival functions were estimated using the Kaplan–Meier method and compared with the log‐rank test. A *p*‐value of <0.05 was considered significant. Analyses were performed using the Statistics Kingdom 2017 software [11].

### Ethics and approval

This observational study was approved by the French Ethics Committee for Infectious Diseases and Tropical Medicine (CER‐MIT) and was declared to the French Data Protection Authority (CNIL, Commission Nationale de l'Informatique et des Libertés, No. 2226468). Written information about the process of data collection to participate was accessible to each patient or their legally authorised representative.

## RESULTS

We compared 8 cases of CAP‐*Ab* with 761 control patients with severe CAP from other pathogens, for which the most frequently isolated micro‐organisms were influenza viruses, followed by *Streptococcus pneumoniae*. No pathogens were found in 30.6% of the cases (*n* = 233). Four cases of CAP‐*Ab* were found in the cohort study, therefore excluded from control patients and already included in the CAP‐*Ab* group. *SARS‐Cov2* was isolated 21 times. A synthesis of causative pathogens is shown in Table 1.

**TABLE 1:** Isolated pathogen in non‐*Ab*‐related CAP (*n* = 761).

Microorganisms	Total
Viruses	242 (31.8)
*Influenza viruses*	150 (19.7)
Other viruses	92 (12.1)
Bacteria	442 (58.1)
*Streptococcus pneumoniae*	98 (12.8)
*Staphylococcus* spp.	83 (10.9)
Panton–Valentine leukocidin‐positive	8 (1.0)
*Haemophilus influenzae*	64 (8.4)
*Klebsiella pneumoniae*	39 (5.1)
Other Enterobacteriaceae	30 (3.9)
*Pseudomonas aeruginosa*	33 (4.3)
*Leptospira* spp.	16 (2.1)
*Legionella* spp.	13 (1.7)
*Stenotrophomonas maltophila*	1 (0.1)
Other bacteria	65 (8.5)
Fungi	17 (2.2)

*Note*: Results are expressed as *n* (%).

Regarding demographics and comorbidities, patients with CAP‐*Ab* had more excessive alcohol use, 75% versus 26%, *p* = 0.005, OR = 8.7 [1.7–43.5] 95% CI (Woolf method). They had a lower body mass index (BMI), median 21.5 [18.5–22] kg/m^2^ versus 25.1 [21.7–30.9] kg/m^2^ (*p* = 0.004), than patients with non‐*Ab*‐related CAP. The vast majority of patients with CAP‐*Ab*, 87.5% (*n* = 7), were smokers. There was no significative difference in terms of diabetes, gender, age, chronic respiratory diseases, or other comorbidities (Table 2A). We found no significant difference in the occurrence of CAP‐*Ab* during the rainy season, respectively, 75% versus 39%, *p* = 0.06 (Table 2A), but we saw a statistical trend that will be discussed.

**TABLE 2a:** Comparison of CAP‐*Ab* and non‐*Ab*‐related CAP.

Parameters	Missing data	CAP‐*Ab*, *n* = 8	Non‐*Ab*‐related CAP, *n* = 761	*p*‐value^a^
Demographics				
Age, y	0	56 [50–59]	62 [52–73]	0.08
Male sex	0	6 (75)	499 (65.6)	0.72
BMI, kg/m^2^	267	21 [18–22]	25 [22–31]	0.004
Rainy season	0	6 (75)	297 (39)	0.06
Comorbidities				
Diabetes mellitus	0	0	261 (34.3)	0.06
Excessive alcohol use	0	6 (75)	195 (25.6)	0.005
Chronic lung disease	0	3 (37.5)	242 (31.8)	0.71
HBP	0	2 (25)	342 (44.9)	0.31
Chronic renal failure	0	0	39 (5.1)	1
Chronic liver disease	0	1 (12.5)	26 (3.4)	0.25
Immunosuppression	0	1 (12.5)	79 (10.4)	0.59
Symptoms in previous days				
Influenza‐like illness^b^	0	2 (25)	272 (35.7)	0.72
Time from symptom onset to presentation, *d*	0	5 [2.5–10.5]	3 [1–6]	0.12
Radiological features				
Lobar consolidation	0	8 (100)	517 (67.9)	0.06
Cavitation	0	0	53 (7.0)	1

*Note*: Data are presented as no. (%) or median [Interquartile range].

Abbreviations: BMI, body mass index; d, day; HBP, high blood pressure; ICU, Intensive Care Unit; y, year.

^a^ Significance *p* < 0.05.

^b^ Influenza‐like illness (ILI) defined by WHO [12]. Chronic renal failure defined as need for haemodialysis. Chronic lung disease is defined as a clinically recorded diagnosis of COPD or bronchiectasis. Chronic liver disease is defined as recorded diagnosis of cirrhosis. Hazardous alcohol use is defined by an AUDIT‐C ≥ 10.

Regarding illness severity, patients with CAP‐*Ab* presented with a higher SAPS II score at admission than patients with non‐*Ab*‐related CAP, median 63 [52.5–77.5] versus 44 [32–58] (*p* = 0.007).

Bacteraemic pneumonia was also more frequent in the CAP‐*Ab* group, 62% (*n* = 5) versus 16% (*n* = 119), *p* = 0.004, OR = 9 [2.1–38.1] 95%CI.

In the CAP‐*Ab* group, mortality was higher, 62.5% versus 24.3%, OR = 5.2 [1.2–21.9] 95% CI, *p* = 0.02, and survival time was shorter with a median of 2 days [1.5–2.5] versus 7 days [3–15], *p* = 0.009 (Table 2B).

**TABLE 2b:** Comparison of CAP‐*Ab* and non‐*Ab*‐related CAP.

Parameters	Missing data	CAP‐*Ab*, *n* = 8	Non‐*Ab*‐related CAP, *n* = 761	*p*‐value*
Severity				
SAPS II score	26	63 [52–77]	44 [32–58]	0.007
Bacteraemia	0	5 (62.5)	119 (15.7)	0.004
Septic shock	47	8 (100)	309 (40.6)	<0.001
Catecholamines	0	8 (100)	415 (54.5)	0.01
Norepinephrine	0	8 (100)	398 (53.3)	0.008
Dobutamine	0	7 (87.5)	85 (11.2)	<0.001
Cardiogenic shock	0	7 (87.5)	121 (15.9)	<0.001
ECMO	0	1 (12.5)	32 (4.2)	0.30
RRT	0	6 (75)	131 (17.2)	<0.001
ARDS	0	8 (100)	516 (67.8)	0.06
Severe ARDS	91	5 (62.5)	177 (23.2)	0.02
PaO_2_/FiO_2_ (nadir), mmHg	91	80 [75.5–105]	153 [100–229]	0.008
Invasive mechanical ventilation	0	8 (100)	477 (62.7)	0.03
Non‐invasive ventilation	0	2 (25)	210 (27.6)	1
High‐flow oxygen therapy	0	4 (50)	137 (18.0)	0.04
Outcomes				
ICU length of stay, d	0	2.5 [2–11]	7 [3–14]	0.14
Length of MV, d	0	2.5 [2–7]	4 [0–11]	0.84
In‐ICU mortality	0	5 (62.5)	185 (24.3)	0.02
Time to death after presentation, d	0	2 [1.5–2.5]	7 [3–15]	0.009

*Note*: Data are presented as No. (%) or median [Interquartile range]. Cardiogenic shock defined according to the American Heart Association [13], septic shock defined according to Sepsis‐3 [14], and severe ARDS means PaO_2_/FIO_2_ ≤ 100 mmHg.

Abbreviations: ARDS, Acute Respiratory Distress Syndrome; ECMO, extracorporeal membrane oxygenation; RRT, renal replacement therapy; SAPS II, Simplified Acute Physiology Score II [15].

Significance *p* < 0.05.

Regarding the need for organ support, significant differences were found in terms of the need for renal replacement therapy (75% vs. 17%, *p* < 0.001), catecholamine use (100% vs. 54%, *p* = 0.01), and the need for invasive mechanical ventilation (100% vs. 63%, *p* = 0.03).

We have a significative difference between our two groups, in propensity of septic shock (100%. vs 40.6%, *p* < 0.001) and in severe ARDS, meaning PaO_2_/FiO_2_ ≤ 100 mmHg (62.5% vs 23.2%, *p* = 0.02).

To note, a significant propensity of cardiogenic shock in the CAP‐*Ab* group (87.5% vs. 11.2%, *p* < 0.001).

A synthesis of those data is shown in Table 2B.

**FIGURE 1:**
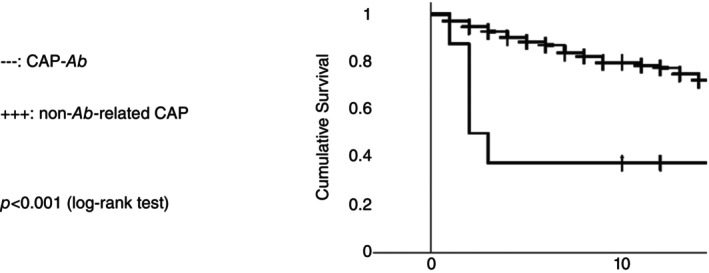
Kaplan–Meier curves [51].

Survival functions are shown in Figure 1.

Laboratory findings on ICU admission showed a propensity to leukopenia (G/L), median 2.15 versus 11.9 (*p* = 0.001) and elevated lactatemia, median 4.4 [3.05–6.2] mmol/L versus 2 [1.3–3.7] mmol/L (*p* = 0.02).

A large majority of patients with CAP‐*Ab*, 75.0% (*n* = 6), had thrombopenia on admission, with a significant propensity to low platelet count compared to patients with non‐*Ab*‐related CAP (median 103 [82.5–144] G/L versus 187 [126–268], *p* = 0.02).

Laboratory findings are summarised in Table 3.

**TABLE 3:** Laboratory findings on ICU admission.

Parameters	Missing data	CAP‐*Ab*, *n* = 8	Non‐*Ab*‐related CAP, *n* = 761	*p*‐value*
Haemoglobin, g/dL	56	13 [10.35–14]	11.9 [10–13.4]	0.59
Absolute leukocyte count, G/L	22	2.15 [0.57–3.84]	11.9 [7.6–16.6]	0.001
Platelet count, G/L	56	103 [82.5–144]	187 [126–268]	0.02
Creatinine levels, μmol/L	12	93 [73.5–129.5]	112 [72–187]	0.40
Bilirubin levels, μmol/L	192	23.5 [9–40.5]	12 [6.5–19]	0.11
Prothrombin time, %	88	65 [46–73]	70 [54–82]	0.39
Lactate levels, mmol/L	49	4.4 [3.05–6.2]	2 [1.3–3.7]	0.02
Creatine phosphokinase levels, mg/dL	336	294 [181–1126]	222.5 [95.5–611]	0.41
Alanine aminotransferase, UI/L	89	36 [31.5–60.5]	30 [17–57]	0.29
Troponin levels, ng/mL	188	23 [17–370]	39 [16–122]	0.997

*Note*: Data are presented as median [interquartile range].

*Significance *p* < 0.05.

Antibiotic susceptibility testing on the eight *Ab* isolates showed that all (100%) were sensitive to ceftazidime, cefepime, piperacillin‐tazobactam, ciprofloxacin, gentamicin, and imipenem. Many (6 of 8, 75%) were also sensitive to ticarcillin, piperacillin and cotrimoxazole. For more details on this issue, we address our readers to our previously published case series report [7].

## DISCUSSION

This study is the first to compare severe community‐acquired *Acinetobacter baumannii* pneumonia (CAP‐*Ab*) with non‐*Ab*‐related severe CAP cases in France.

Regarding non‐*Ab*‐related CAP, our data seems consistent with the literature on severe CAP, for example, on septic shock proportion (49.4%), mortality (19%), or need for RRT (16.2%) [16]. In addition, the pre‐eminence of viruses in CAP, especially influenza and picornaviruses, is now more extensively described since respiratory multiplex PCR is used for the diagnosis [5, 9, 17, 18, 19].

Regarding CAP‐*Ab*, we found several significative differences from non‐*Ab*‐related CAP. First, we found the pre‐eminence of excessive alcohol consumption, which is widely described in the literature [1, 2, 3, 4, 20, 21, 22, 23, 24, 25], with the notable exception of the retrospective study by Ong et al. [26] Excessive alcohol use has been known to be a poor prognostic factor in CAP [27]. However, we did not find any statistically significant differences in terms of diabetes and chronic lung disease, though this could be due to lack of diagnosis in our CAP‐*Ab* patients or regional variation in risk factors as proposed by Dexter et al. [4] Our study appears to be the first to highlight a statistically significant difference with a lower BMI, which appears to be consistent with chronic alcoholism and hypalbuminaemia [20]. The seasonal influence of the warmer months of the year was not confirmed using the Fisher test, due to our limited sample size in CAP‐*Ab* group, if we consider the literature on this topic [1, 2, 3, 4, 20, 21, 22, 23, 24, 25, 26]. Nevertheless, our data suggest a statistical trend on this topic.

The lethality rate observed in both groups (62.5% vs. 24.3%, *p* = 0.02), is consistent with the literature on CAP and CAP‐*Ab* [2, 3, 4, 16, 21, 22, 23, 24, 26, 28], except for the work by Davis et al., where they found that a local antibiotic protocol reduces CAP‐*Ab* mortality to 11% [20]. This high lethality rate cannot be explained by a high resistance pattern of *Ab* isolates. Five of our eight isolates were indeed wild‐type strains, and the rest have a low resistance pattern, as shown in other papers [20, 26, 29, 30]. The clinical presentation of CAP‐*Ab* appeared to fit the description by Leung et al., which is a fulminant course [3]. Indeed, median time to eventual death in the CAP‐*Ab* group was significantly shorter (2 vs. 7 days, *p* = 0.009).

In our series, patients with CAP‐*Ab* always required catecholamine use for septic shock. The literature found lower rates of septic shock between 58% and 92% [3, 20, 21, 26].

The significant propensity of cardiogenic shock in the CAP‐*Ab* group (87.5%), justifying the use of dobutamine, had not yet been described. We diagnose cardiogenic shock with bedside ultrasound examination, according to the American Heart Association [13], as a cardiac index lower to 2.5 L/s/m^2^, which was inappropriate in the context of vasoplegic shock. It was difficult to distinguish between septic cardiomyopathy or cardiac decompensation. Yet, none of the CAP‐*Ab* patients had dilated heart disease, which is the most common pattern in alcoholic cardiomyopathy. To our knowledge, only one study has described cardiogenic shock in patients with CAP‐*Ab* [2]. The increased use of bedside ultrasound examination could in the future result in the more frequent observation of cardiogenic shock in patients with severe CAP‐*Ab*. We note that we had found no differences in troponin levels on admission between our two groups. That could be due to a delayed increase in troponin levels. In the setting of cardiogenic shock, it is not recommended to wait for the presence of elevated cardiac enzymes before initiating treatment [13]. In the CAP‐*Ab* group, one patient had venoarterial extracorporeal membrane oxygenation in an attempt to treat refractory cardiac shock, but that did not prevent multiple organ dysfunction syndrome.

All CAP‐*Ab* patients required invasive mechanical ventilation for acute respiratory distress syndrome (ARDS). Again, such presentations had been described in literature, generally on a smaller scale, with a need for invasive mechanical ventilation between 72 and 85% [3, 20, 21]. Moreover, severe ARDS (PaO_2_/FiO_2_ < 100 mmHg) was more frequent in the CAP‐*Ab* group (62.5 vs. 23.2%, *p* = 0.02), but extracorporeal membrane oxygenation had not been initiated because patients died due to septic shock and multiple organ failure.

A need for renal replacement therapy (RRT) was strongly correlated with CAP‐*Ab* patients, which is consistent with its severity [2, 26], but such a proportion has not been found in the current literature [3], perhaps because policies for the management of acute renal failure, septic shock, and ARDS vary greatly across ICUs [31, 32].

Regarding laboratory findings, leukopenia and thrombopenia at admission seemed to be more frequent in CAP‐*Ab* patients, as seen in other case reports [3, 20, 22] along with elevated lactatemia, worst prognostic score, such as the SAPS II, and predominant bacteraemia [3, 20]. This last feature has been shown to be a poor prognostic factor in CAP‐Ab [3, 33]. Leukopenia and thrombopenia could be good discriminating factors for CAP‐*Ab* [3], given their low incidence in non *Ab*‐related CAP [16], even with septic shock [28]. Moreover, they could be a prognostic factor in sepsis [3, 28, 34].

We know from our case‐series report [7] that all patients with CAP‐*Ab* received inappropriate first line antibiotic therapy (i.e., a cephalosporin such as ceftriaxone or cefotaxime, and azithromycin, as recommended [35, 36]) despite a low resistance pattern. The importance of early and appropriate antibiotic treatment is known to improve the outcome of bacterial infections [14, 20, 37, 38]. Improving outcomes could require better identification by Gram staining [39] or early identification with new processes [40, 41]. *A. baumannii* could be difficult to detect via Gram‐staining because of its phenotypic variability [1], which could lead to delayed treatment by misidentification. Rapid diagnostic tests (RDTs), such as multiplex real‐time PCR [42, 43, 44, 45], or rapid identification on blood cultures [46, 47] seem more efficient, but cost and availability must be considered [48]. Next‐generation sequencing (NGS) also appears to be a promising technology for identifying this type of pathogen in the near future [41]. Ultimately, improving outcome requires expert advice (infectious disease physicians and pharmacologists) [ 40, 47, 49] and perhaps individual protocols for at‐risk patients during the rainy season [20, 50]. Considering the susceptibility of *A. baumannii* strains in CAP, that have been shown to not display a very resistant pattern [ 7, 20, 26, 29, 30], we suggest using cefotaxime with ciprofloxacin as empirical therapy if CAP‐*Ab* is suspected.

The main limitations of our study are its retrospective nature and the limited number of CAP‐*Ab* cases. Also, our comparison did not match the same period, regarding our two groups, but we have considered that it gave a relevant preview of the specificities of CAP‐*Ab*.

## CONCLUSION

CAP‐*Ab* presents itself as a fulminant pneumonia, associated with multiple organ failure, such as ARDS and septic cardiomyopathy, and burdened with high mortality (62.5%). Patients with CAP‐*Ab* seem more likely to experience excessive alcohol use and malnutrition. Leukopenia and thrombopenia at admission could be discriminating factors for CAP‐*Ab*. Such conditions could be related to this pathogen and prompt a broader spectrum of antibiotic therapy for *A. baumannii*, in order to improve patient outcomes.

## AUTHOR CONTRIBUTIONS

**Axel de Mangou**: Data conception; acquisition; analysis; interpretation. **Agathe Combe**: Data conception; acquisition; analysis; interpretation. **Amelie Renou**: Data conception; interpretation. **Chloe Combe**: Conception. **Radj Cally**: Conception. **Marie Lagrange‐Xelot**: Data conception; interpretation. **Nicolas Allou**: Data conception; interpretation and design of the work; writing—draft; writing—review. **Guillaume Miltgen**: Data conception; interpretation and design of the work; writing—draft; writing—review. **Charles Vidal**: Data conception; interpretation and design of the work; writing—draft; writing—review.

## CONFLICT OF INTEREST STATEMENT

The authors have no conflict of interests to disclose.

## Data Availability

The datasets used and/or analyzed during this study are available from the corresponding author on reasonable request.
